# Semi‐Transparent, Pixel‐Free Upconversion Goggles with Dual Audio‐Visual Communication

**DOI:** 10.1002/advs.202302631

**Published:** 2023-09-22

**Authors:** Chun‐Jen Shih, Chao‐Yang Lin, Kai Chen, Nurul Ridho Al Amin, Dian Luo, I‐Sheng Hsu, Abdul Khalik Akbar, Sajal Biring, Chih‐Hsuan Lu, Bo‐Han Chen, Shang‐Da Yang, Jiun‐Haw Lee, Shun‐Wei Liu

**Affiliations:** ^1^ Graduate Institute of Photonics and Optoelectronics and Department of Electrical Engineering National Taiwan University Taipei 10617 Taiwan; ^2^ Organic Electronics Research Center and Department of Electronic Engineering Ming Chi University of Technology New Taipei City 24301 Taiwan; ^3^ Robinson Research Institute, Faculty of Engineering Victoria University of Wellington Wellington 6012 New Zealand; ^4^ MacDiarmid Institute for Advanced Materials and Nanotechnology Wellington 6012 New Zealand; ^5^ The Dodd‐Walls Centre for Photonic and Quantum Technologies Dunedin 9016 New Zealand; ^6^ Institute of Photonics Technologies National Tsing Hua University Hsinchu 300044 Taiwan

**Keywords:** infrared visualization, optical communication, organic upconversion devices, pump‐probe spectroscopy, wearable electronics

## Abstract

The intractable brittleness and opacity of the crystalline semiconductor restrict the prospect of developing low‐cost imaging systems. Here, infrared visualization technologies are established with large‐area, semi‐transparent organic upconversion devices that bring high‐resolution invisible images into sight without photolithography. To exploit all photoinduced charge carriers, a monolithic device structure is proposed built on the infrared‐selective, single‐component charge generation layer of chloroaluminum phthalocyanine (ClAlPc) coupled to two visible light‐emitting layers manipulated with unipolar charges. Transient pump‐probe spectroscopy reveals that the ClAlPc‐based device exhibits an efficient charge dissociation process under forward bias. This process is indicated by the prompt and strong features of electroabsorption screening. Furthermore, by imposing the electric field, the ultrafast excited state dynamic suggests a prolonged charge carrier lifetime from the ClAlPc, which facilitates the charge utilization for upconversion luminance. For the first time, >30% of the infrared photons are utilized without photomultiplication strategies owing to the trivial spectrum overlap between ClAlPc and the emitter. In addition, the device can broadcast the acoustic signal by synchronizing the device frequency with the light source, which enables to operate it in dual audio‐visual mode. The work demonstrates the potential of upconversion devices for affordable infrared imaging in wearable electronics.

## Graphical abstract

This work proposes a dual audio‐visual infrared communication technology based on the singe‐pixel, large‐area, semi‐transparent organic upconversion device. While the device brings the high‐resolution (maximum 5,086 pixels per inch) invisible infrared images (780 nm) to sight (523 nm) without complex photolithography technique, it also broadcast the melody by synchronizing the device with the signal frequency.

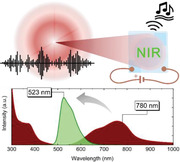

## 1 Introduction

Mammals find restricted vision outside the visible spectra, which is typically 400–700 nm in wavelength for human beings. It is formidable to go beyond the limit by extending human sensation into infrared frequency,^[^ ^1^ ^]^ finding alluring possibilities in night vision,^[^ ^2^ ^]^ food safety,^[^ ^3^ ^]^ image fusion,^[^ ^4^ ^]^ machine learning,^[^ ^5^ ^]^ defect detection,^[^ ^6^, ^7^ ^]^ and oncology,^[^ ^8^, ^9^ ^]^ to name a few. To achieve this, researchers have devoted themselves to improving the performance of complementary metal‐oxide‐semiconductor (CMOS) by incorporating potential infrared sensing materials.^[^ ^10^, ^11^ ^]^ However, previous demonstrations on CMOS image sensors required delicate photolithography techniques to advance pixel scaling without sacrificing crosstalk, color filters to acquire spectral differentiation, wafer bonding to support external electrical readout, and buffer layer to tackle the irreconcilable contact barriers between heterogeneous compounds.^[^ ^12^, ^13^ ^]^ Challenges call for originality in a brand‐new design to address the soaring demand for an affordable visualization system in large‐area, low‐intensity infrared image applications.^[^ ^14^ ^]^

Infrared‐to‐visible upconversion device, an alternative approach that circumvents costly pixel scaling conventions in mainstream technology, releases high‐resolution infrared images from complex readout integrated circuits.^[^ ^15^, ^16^ ^]^ In addition to the compatibility combined with the existing compact lens set,^[^ ^17^ ^]^ the upconversion device offers high flexibility of monolithically stacking various emission layers (EML) on top of the infrared‐sensitive charge generation layer (CGL).^[^ ^18^ ^]^ Although disordered organic light‐emitting diodes (OLEDs) were expected to alleviate the lattice mismatch issue of heterogeneous compound semiconductors in the first place,^[^ ^19^, ^20^ ^]^ previous studies suffered barely satisfactory luminance in return. Researchers have since investigated other potential infrared‐sensitive functional materials, screening colloidal quantum dots,^[^ ^21^, ^22^ ^]^ organic‐inorganic hybrid halide polycrystals,^[^ ^23^ ^]^ and non‐fullerene acceptors.^[^ ^24^, ^25^ ^]^ Still, limited success was received in terms of photon‐to‐photon upconversion efficiency (η_*p* − *p*_),^[^ ^26^ ^]^ even leveraging more light‐emitting subunits.^[^ ^27^, ^28^ ^]^ Decent efficiency was fulfilled only after a three‐terminal configuration of the high‐gain phototransistor,^[^ ^21^, ^29^ ^]^ yet it complicated the fabrication process and limited the dynamic response range. We argue that high utilization of infrared photons can be achieved in a two‐terminal structure after regulating both types of charge carriers from the single‐component chloroaluminum phthalocyanine (ClAlPc).^[^ ^30^ ^]^

## 2 Results and Discussion

### 2.1 Design Principles and Operational Mechanism

**Figure 1:**
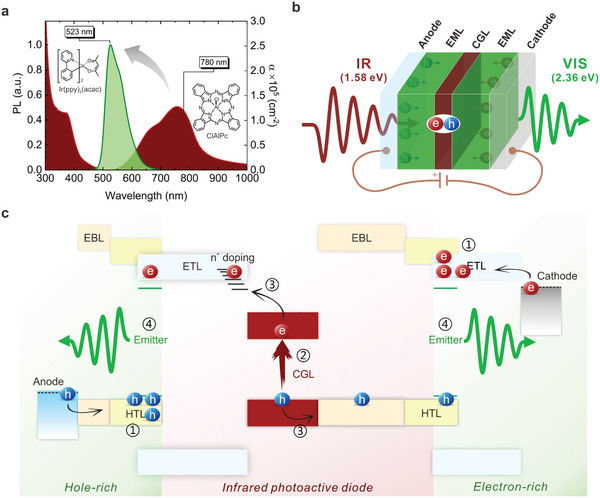
Schematic illustration of infrared upconversion technology. a) The absorption coefficient (α) spectrum of the ClAlPc solid‐state thin film and the photoluminescent (PL) spectrum of the Ir(ppy)_2_(acac) emitter dispersed in BCzPh: CN‐T2T host matrix. b) Cross‐sectional profile of the device stacking, sandwiching charge generation layer (CGL) between two visible light emission layers (EML). IR, incident infrared stimulus. VIS, upconversion visible luminance. c) Energy band diagram of the device in the presence of infrared signal excitation (flat band potential). Each block represents the frontier orbitals of the layer (not to scale). ①–④ guides the upconversion process in sequence. ETL/ HTL, electron/ hole‐transporting layer. EBL/ HBL, electron/ hole‐blocking layer.

We target the low‐energy Q‐band of the ClAlPc with the monochromatic 780 nm near‐infrared wavelength (1.59 eV in photon energy) (**Figure** **1**a). The symmetric disc‐like macrocyclic centering of the Al^+^‐Cl^−^ vertical dipole satisfied the intramolecular excited‐state polarization upon photoexcitation,^[^ ^30^ ^]^ which sets it apart from the common organic materials featuring a low dielectric constant. Detailed steady‐state photophysics of solid‐state ClAlPc diluted in an inert matrix was demonstrated (Figure S1, Supporting Information). Note that unlike the nonlinear short‐range mechanisms devising intermediate excited states for anti‐Stokes shift (e.g., lanthanide‐doped nanoparticles^[^ ^31^ ^]^ and triplet–triplet annihilation^[^ ^32^ ^]^), we develop the upconversion process on different long‐range molecules sharing the responsibilities. The upconversion green spectrum is released from the bis(2‐phenylpyridine)iridium(III)‐acetylacetonate [Ir(ppy)_2_(acac)] phosphorescent emitter with the charge carriers supported by the infrared photoactive ClAlPc.

To recover the loss in most upconversion devices managing single‐type charge carriers, we monolithically construct a two‐terminal organic upconversion device (OUD) to consume both types of polarons from the CGL, that is, electron‐hole pairs are completely depleted without carrier loss (Figure 1b). Instead of developing bulk heterojunction interfaces for optimal exciton dissociation,^[^ ^33^ ^]^ single‐component ClAlPc neat film served as the CGL without introducing any fullerene derivatives in this work. Therefore, the re‐absorption loss can be mitigated by minimizing the spectral overlap between the two main molecules^[^ ^28^ ^]^ (cf. Figure 1a). Besides, all photoinduced charge carriers can be entirely ascribed to the specific ClAlPc for clarity. We design device integration consuming ambipolar charge carriers based on the band diagram depicted in Figure 1c. The device is driven under a forward bias throughout the study (ITO as the anode and aluminum as the cathode). While the first EML is operated at a hole‐rich condition expecting electron arrival, the second EML is vice versa managing an electron‐rich situation (①). Once the desired infrared stimulation triggers the CGL (②), photoinduced charge carriers that experience the Stark screening effect are guided towards respective EMLs (③) right after a self‐dissociation process,^[^ ^30^ ^]^ which will be elaborated in the pump‐probe spectroscopy later on. To conquer the substantial energy barrier between CGL and the first EML, n‐type doping of the electron‐transporting layer interface is deployed to assist charge hopping.^[^ ^34^, ^35^ ^]^

The delocalized charge carriers are anticipated to support a high‐energy emissive spectrum from the phosphorescent emitter dispersed in an exciplex co‐host blend (④).^[^ ^16^, ^36^ ^]^ In addition to the large energetic offset regulating the space charges at the interface, the homogeneous, balanced hopping routes blending charge‐transporting layers as the bipolar co‐host facilitate the recombination process.^[^ ^37^, ^38^ ^]^ Hence, a barrier‐less charge supply with a driving voltage approaching the emitter bandgap can be imagined,^[^ ^16^, ^39^ ^]^ accessing high charge carrier utilization for upconversion luminescence. In fact, a sub‐gap turn‐on voltage lower than the photon energy of the emitter was observed on account of the photovoltaic absorber.^[^ ^23^ ^]^ Here, we emphasize the fundamental difference in charge generation mechanism between photoactive OUD and tandem OLED.^[^ ^28^ ^]^ The upconversion process is initiated only after the infrared stimulus of interest, which differs from the field‐induced charge generation at the heterojunction interface of tandem OLEDs, especially in the dark.^[^ ^40^ ^]^

### 2.2 All‐Organic Upconversion Devices

**Figure 2:**
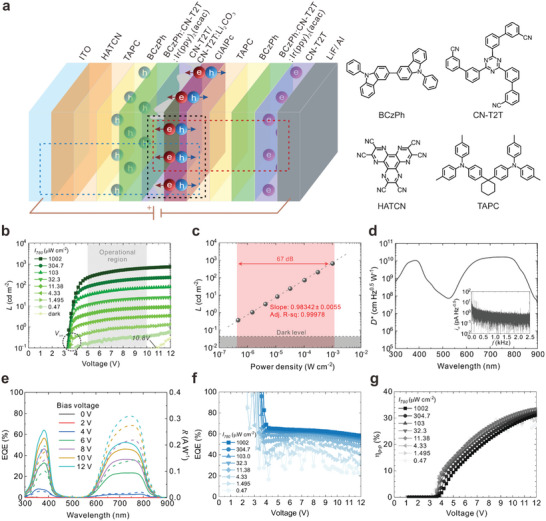
Infrared sensing utilizing bipolar charge carriers. a) Cross‐sectional view of the device stacking (not scale to size between layers). The molecular structure of the charge‐transporting materials is presented on the right. More details on the infrared sensing ability (enclosed by a black dash line) and unipolar OUDs managing single‐type charge carriers (red and blue dash lines) are available in Supporting Information. b) Bias‐dependent upconversion luminance (*L*) of the device under various infrared power densities. *V*_on_ stands for turn‐on voltage. c) Upconversion luminance of the device driven at 10.0 V under various infrared power densities (black spheres). The red area demonstrates the linear fitting result—the adjusted R square value describes the mean square deviation of the fitting. The grey dash line is the ideal case guide for the eye. The shaded grey level tells the leakage luminance under 10.0 V without an infrared stimulus. d) Specific detectivity (D*) spectra of the device driven at 10.0 V. Inset shows the noise current (*i*_n_) of the device recorded at the same bias. e) External quantum efficiency (EQE, solid lines) and responsivity (*R*, dash lines) spectra of the device under various bias voltages. f) Bias‐dependent electroluminescent EQE of the device under various infrared power densities. The large deviations approaching *V*_on_ are excluded for clarity. g) Bias‐dependent photon‐to‐photon upconversion efficiency (η_p − p_) of the device under various infrared power densities.

The bipolar OUD managing both types of charge carriers were created and characterized accordingly (**Figure** **2**a, Experimental Section). Given the optical interference stacking cavity length stands out in multilayered structures, the optimized device demonstrates an appropriate resonant position for incident infrared irradiation according to the transfer matrix model (Figure S2, Supporting Information). It suggests that the device acts with precise wavelength selectivity and trivial re‐absorption loss for visible light out‐coupling. As recorded in Figure 2b, the device followed an explicit photo‐switching response with commensurate upconversion luminance in the bias voltage range of 5.0–10.0 V, differentiating the infrared power density down to sub‐microwatts per centimeter square. The device turned on at the bias voltage of ≈3.2 V (V_*on*_ defined at 0.1 cd m^−2^) depending on the incident infrared intensity, which is about twice the value of the device managing single‐type charge carriers (Figures S3 and S4, Supporting Information). In the absence of an infrared stimulus (denoted as dark), no discernible light (<0.1 cd m^−2^) was recorded until the bias voltage of 10.8 V. Indeed, under a bias voltage of 10.0 V, the device could track the light intensity stretching more than three orders of magnitude monotonically (Figure 2c), accounting for a 67 dB of linear dynamic range. The effect of the leakage current on the upconversion luminance was shaded in grey for reference.

We further probed the photosensitivity of the device with respect to the weak infrared stimulus. The device demonstrated a specific detectivity of 1.56 × 10^10^ Jones (cm Hz^−1/2^ W^−1^) at the wavelength of 780 nm (Figure 1d). We believe the reason governing our specific detectivity is the noise aroused by the EMLs. Without introducing the EMLs, the ClAlPc CGL could maintain a noise level of several fA Hz^−1/2^ (Figure S5, Supporting Information). The spectral response of the entire upconversion device is recorded in Figure 2e. The device followed a precise selectivity toward the incident light wavelength in line with the absorption spectrum of the ClAlPc neat film (cf. Figure 1a). Imposing a more substantial external bias amplified the representative transition bands with a saturated EQE of ≈50% while retaining limited response in the visible spectrum. On the other hand, the electroluminescent efficiency of the whole upconversion device is recorded in Figure 2f. A nearly doubled electroluminescent efficiency was received in terms of EQE compared to the device exploiting single‐type charge carriers (Figure 2f; see Figures S3e and S4e, Supporting Information), verifying our strategy of recovering the loss in most upconversion devices consuming single‐type of charge carriers. The moderate efficiency roll‐off under high bias voltage implies the efficient charge carrier confinement of the exciplex co‐host system benefitted from the sizeable energetic offset, reinforcing the alignment between the recombination zone and the emission layer constructed.

Overall, the bipolar OUD achieved an efficient infrared‐to‐visible upconversion efficiency (η_*p* − *p*_, the metric that evaluates the infrared photon utilization rate for visible light emission) following the concept of harnessing both types of photoinduced charge carriers from the single‐component ClAlPc CGL (Figure 2g). The η_*p* − *p*_ was determined by the upconversion luminance retrieved from Figure 2b (More information on the calculation process can be referred to Experimental Section). The strong correlation between the η_*p* − *p*_ and the external bias substantiates that the charge carrier supplied by the CGL dominates the upconversion process on account of minor electroluminescent efficiency roll‐off (Figure 2f). A maximum η_*p* − *p*_ of ≈27.49–31.20% was recorded at 10.0 V, depending on the incident intensity, which is among the highest reported values (**Table** **1**). In particular, the photoinduced charge carriers contributed by the CGL were fully utilized without introducing photomultiplication strategies,^[^ ^21^, ^28^, ^29^ ^]^ with a twofold electroluminescent efficiency thanks to the synergistic effect between the CGL and EMLs. Note that this work demonstrates the superiority of the OUD based on device integration, realizing an unprecedented quantum yield comparable to that of the nonlinear upconversion approaches.^[^ ^41^, ^42^ ^]^

**Table 1:** Summary of the key parameters on the notable upconversion devices.

Infrared photoactive system^a)^	Visible light‐emitting system	*V_on_* ^b)^ [voltage]	LDR^c)^ [dB]	*η_p__‐p, max_* [%]	Active area^d)^ [cm^2^]	Reference
PbS CQDs (940 nm, 10 mW cm^−2^)	CdSe/ ZnS QDs	≈4	≈60 (≈1 mW cm^−2^)	6.5@10V	0.05	[22]
FAPbI_3_ (830 nm, 32 mW cm^−2^)	CBP: Ir(ppy)_3_	<2.5	24 (0.94 µW cm^−2^)	3@5V	1	[23]
PTB7‐Th:IEICO‐4F:PC_71_BM (895 nm, 0.464 mW cm^−2^)	CBP:Ir(ppy)_2_(acac)	1.6	84.4 (3.2 µW cm^−2^)	12.9@8V	0.1	[25]
PTB7‐Th:COTIC‐4F (940 nm, 0.1038 mW cm^−2^)	BCzPh:CN‐T2T: Ir(ppy)_2_(acac)	2.0	78 (0.75 µW cm^−2^)	12.56@8V	10.35	[26]
PDPP3T:PC_61_BM (850 nm, <1 mW cm^−2^)	Be(pp)_2_: Ir(ppy)_2_(acac)	≈2.8	≈56 (≈3 µW cm^−2^)	29.6@12 V	4	[28]
Polymer donor:PC_71_BM (1050 nm, 4.2 mW cm^−2^)	Alq_3_	NA	NA	0.15@3V	2	[61]
D8‐Cl:Y6:PC_71_BM (850 nm, 0.7 mW cm^−2^)	CBP: Ir(ppy)_2_(acac)	≈1.5	61.6 (1.4 µW cm^−2^)	19.16@8V	0.1	[62]
ClAlPc (780 nm, 32.3 µW cm^−2^)	BCzPh:CN‐T2T: Ir(ppy)_2_(acac)	3.2	67 (0.47 µW cm^−2^)	31.2@10V	7.9	This work

^a)^ The wavelength and power density of the incident infrared signal are noted in parentheses;

^b)^ The turn‐on voltage is defined as the voltage when the device shows 0.1 cd m^−2^;

^c)^ The linear dynamic region. The minimum power density of the infrared stimulus while keeping linear upconversion luminance is noted in parentheses;

^d)^ The maximum size of the sample demonstrated in the work.

### 2.3 Transient Photoexcited Analysis

The underlying mechanism responsible for the record‐high upconversion efficiency was further investigated by exploring the transient response of the ClAlPc. The ultrafast photoexcitation dynamics, a general phenomenon in organic semiconductors, provide insights into the photophysics of the device under operation. Here, we leveraged the ultrafast pump‐probe spectroscopy on the single‐component ClAlPc with an organic photodetector (OPD) structure.^[^ ^30^ ^]^ Since the device is operated under forward bias, its ultrafast behavior is of high interest when compelling an external electric field; therefore, we conducted ultrafast pump‐probe spectroscopy (reflection mode) on the device under zero bias and forward bias to probe the ultrafast optical response of the device correlated with the field. The pump‐probe spectroscopy was also conducted on single‐layer ClAlPc and bilayer structures of ClAlPc/ TAPC (transparent mode) to study their photophysics under NIR photoexcitation (see Supporting Information). The experiment setup was built on double pass multiple plate compression (DPMPC) based on a ytterbium‐based laser.^[^ ^43^ ^]^ More experiment details on transient absorption (TA) spectra map can be found in Supporting Information.

**Figure 3:**
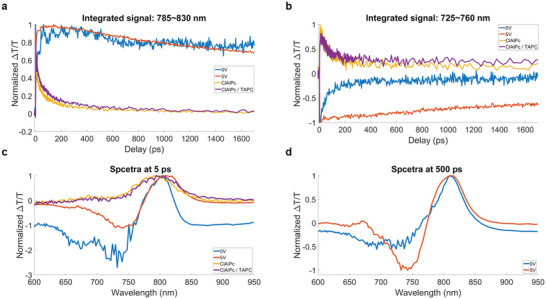
Pump‐probe spectroscopy measurement result of ClAlPc OPD and thin film samples. a,b) Normalized kinetics of the OPDs (external bias at 0 and 5 V) and the thin film samples (single layer ClAlPc and bilayer ClAlPc/TAPC). The curves are obtained by integration over a certain wavelength range, for example, 785–830 nm in (a), and 725–760 nm in (b). In (b), the signals of the device under bias are normalized to the negative maxima, while the signals of ClAlPc and ClAlPc thin film samples are normalized to their positive maxima. c) Normalized TA change spectra (Δ*T*/*T*) at a given delay of 5 ps. d) Normalized TA change spectra (Δ*T*/*T*) of the device at 0 and 5 V at a given delay of 500 ps.

For ClAlPc and ClAlPc/TAPC thin film samples coated on glass substrates, we note ground state bleaching of singlet excitons at ≈785 nm (**Figure** **3**c), with a short lifetime of <50 ps (Figure 3a), corresponding to the absorption peak of ClAlPc.^[^ ^44^ ^]^ The bilayer ClAlPc/TAPC displays similar kinetics and spectra to ClAlPc (Figure 3a,c), suggesting the absence of energy or charge transfer between ClAlPc and TAPC within the bilayer structure.

For the unbiased OPD device (blue lines), we observe positive TA signals ≈785–830 nm (Figure 3a) and negative TA signals ≈725–760 nm (Figure 3b). The signal lifetime and early time spectra (Figure 3a–c) differ from those of ClAlPc and ClAlPc/TAPC (yellow and purple lines) thin film samples and cannot be simply explained as the feature of singlet exciton generation. The spectral evolution in early time (<100 ps) can be related to the exciton dissociation,^[^ ^45^, ^46^, ^47^, ^48^, ^49^ ^]^ intrinsic exciton recombination,^[^ ^50^ ^]^ formation of charge transfer state,^[^ ^47^, ^51^, ^52^ ^]^ and screening of electroabsorption.^[^ ^46^, ^47^, ^50^, ^52^, ^53^, ^54^, ^55^, ^56^, ^57^ ^]^ After 100 ps (Figure 3d; Figure S7c, Supporting Information), the device TA spectra maintain a long‐lived signature, in contrast to the short‐lived signals of the ClAlPc and ClAlPc/TAPC thin film samples (Figure S7a,b, Supporting Information). We interpret these features as the signatures of the long‐lived free‐charge carriers, which resemble the photovoltaic characteristics of the OPD device.^[^ ^30^ ^]^

For the forward bias case at 5 V (orange lines), the same polarity as the upconversion device, we observe long‐lived positive signals ≈785–830 nm (Figure 3a) and negative signals ≈725–760 nm (Figure 3b) in the TA map. These spectral characteristics (Figure 3c,d) mirror the persistent TA spectrum of the OPD device under zero bias, implying that photocurrent generation is taking place. By closely examining the long‐lived TA spectrum (Figure 3d), we observe a spectral shape resembling the inverse of first derivative of the ClAlPc absorption spectra. This suggests that the observed spectral features are related to electroabsorption.^[^ ^55^, ^56^ ^]^ Under external bias, the absorption spectra of ClAlPc are redshifted owing to the Stark effect (Figure S9a, Supporting Information).^[^ ^58^, ^59^ ^]^ When the device is exposed to an infrared stimulus, the external bias assists in the dissociation of charge carriers in ClAlPc.^[^ ^30^ ^]^ This process screens out the external field and subsequently shifts the absorption spectrum towards the unbiased absorption spectrum (Figure S9b, Supporting Information). Consequently, this effect results in a decrease in absorption above and an increase in absorption below 785 nm,^[^ ^53^, ^55^, ^56^ ^]^ a wavelength position near the ClAlPc absorption maxima. Since the field screening appears immediately after the photoexcitation (Figure S8d, Supporting Information), we attribute the overall features to the prompt photoinduced free charge generation under external forward bias. The inverse of the first derivative of ClAlPc absorption spectra shape remains clear in late time (Figure 3d), suggesting the long‐lasting field screening and, therefore, the long free charge lifetime.

It is worth noting that the pump‐probe experiments were carried out under identical conditions, except for the presence of external bias. The high absolute values of TA signals from the OPD with 5 V forward bias (Figure S7d, Supporting Information) indicated a stronger photo response due to the combination of stronger electroabsorption effect^[^ ^57^, ^60^ ^]^ and the higher photocurrent generation efficiency^[^ ^30^ ^]^ when compared to the device with zero bias (Figure S7c, Supporting Information). Our findings indicate that ClAlPc active layer demonstrates immediate and efficient photocurrent generation in the single‐component device structure under infrared excitation. More strikingly, the device structure and external bias operation prolong the lifetime of the free‐charge carriers. These two phenomena directly contribute to the efficient charge utilization from the ClAlPc active layer and, therefore, the high quantum efficiency of the upconversion device.

### 2.4 Infrared Signal Transmission

Although the capability of transforming invisible low‐energy infrared photons into the visible spectrum has been identified, whether the pixel‐free upconversion device can serve as a suitable infrared transmission technology for extending human sensation is still under debate. Given the straightforward visualization process of the upconversion device without introducing bulky readout integrated circuits for pixelation layout, most demonstrations have focused on the optical imaging performance of upconversion devices previously, including the metrics of upconversion efficiency, spatial resolution, and signal‐to‐noise ratio. While optical imaging is indispensable for human visual awareness, the electronic signal can deliver high‐bandwidth transmission for back‐end data processing and storage. The electronic output of the upconversion devices remains unexplored for high‐frequency signal transmission.^[^ ^61^, ^62^ ^]^

**Figure 4:**
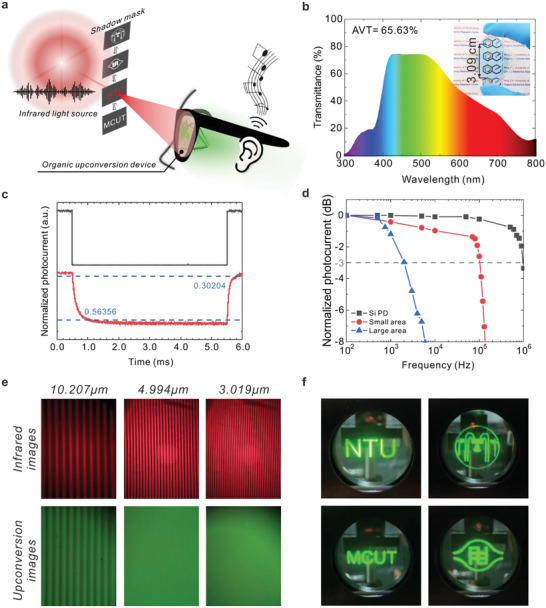
Infrared signal transmission on large‐area, semi‐transparent, upconversion goggles. a) Wireless dual‐mode operation of the infrared images defined by the shadow mask (DC component) and audio signal triggering the light source (AC component) on OUDs. The invisible infrared signal is rendered in red for demonstration. b) The transmittance spectrum of the semi‐transparent OUD. Inset shows the actual photo of the device with an active area of 7.9 cm^2^. c) Time‐resolved normalized photocurrent of the device (red) relative to a 100 Hz modulated infrared light signal (780 nm; −1.0 mW cm^−2^; black). d) Normalized photocurrent as a function of modulating frequency with different size area of the OUD. The response speed of a standard silicon photodiode (818‐SL, Newport) is included for reference. e) Photographs of infrared light propagating through line‐shaped photomasks with three different line spacing widths and the visible image restored by the semi‐transparent device. f) Photograph of the infrared images revealed by the see‐through upconversion goggles.

Here, we demonstrate a close‐eye user interface that operates a downlink‐modulated infrared signal in dual audio‐visual mode (**Figure** **4**a). As the upconversion goggles could bring the infrared image defined by the shadow mask into sight for visual sense, the audio signal was transmitted by modulating the light source intensity (frequency) simultaneously. The device received the input audio message by synchronizing the device with the light source signal. To bring out the lightweight, see‐through, and process flexibility in disordered optoelectronics, we showcased compact infrared visualization goggles built on large‐area, semi‐transparent, single‐pixel OUD devices (inset in Figure 4b). By replacing the opaque top electrode with the transparent Cu: Ag alloy thin film,^[^ ^63^ ^]^ the overall device stacking preserved a high transmittance in the visible spectrum, accounting for a 65.63% average visible transmittance (*AVT*) regarding the transmittance weighted by human photoreceptors. Note that we demonstrate a proof‐of‐concept prototype on achromatic glass substrates with no curvature. It would require delicate optical engineering to match the unique visualization system of human vision. To examine the feasibility of the device as a high‐frequency signal receiver, we applied a square wave‐modulated infrared signal to record the temporal response of the semi‐transparent device (Figure 4c). The large‐area OUD tracked a real‐time infrared signal (frequency of 100 Hz) with a rise/ fall time of 563.56/ 302.4 µs, which is comparable to that of upconversion device based on lead sulfide quantum dots.^[^ ^64^ ^]^ Although it is considered fast enough and beyond the response speed of human vision, we believe the factor governing our response speed is the sample size, that is, the expanded active area increases the capacitance‐resistance time constant.^[^ ^65^ ^]^ Indeed, the small‐area device (4 mm^2^) reached a −3 dB bandwidth of 100 kHz compared to the large‐area device (2 kHz) in Figure 4d. Nevertheless, the melody (Canon in D major) was explicitly broadcast by the large‐area goggles in connection with a speaker (The system setup can be found in Figure S11, Supporting Information). The full demonstration of the audio signal transmission can be found in Video S1 (Supporting Information). Note the infrared signal was modulated by the melody directly for the sake of simplicity. The transmitted signal can be encrypted with communication protocols addressing privacy concerns. To evaluate the spatial resolution of the device, we defined the infrared pattern by propagating through the narrow spacing of the line‐shaped photomasks (Fong Cheng Cam Tech. Co., LTD). The actual size of the spacing width was ascertained in Figure S12 (Supporting Information) with 10.207 ± 0.071, 4.994 ± 0.115, and 3.019 ± 0.137 µm, respectively. As demonstrated in Figure 4e, clear infrared line patterns with three different spacing widths can be discovered. However, the line pattern was expanded after the upconversion process experienced the lateral current spreading issue.^[^ ^20^ ^]^ By downscaling the spacing size, two green line stripes may overlap and could not be distinguished eventually. In our case, the upconversion image of the 3.019 µm spacing width photomask could barely be identified. Blurred line pairs were found only at the border with better contrast. However, a uniform line‐shaped upconversion pattern was discovered from the 4.994 µm photomask, corresponding to an image resolution of ≈5086 pixels per inch. Note that it required only a 2 K resolution of foveated display considering the visual acuity of ≈10 degrees field of view for human eyes.^[^ ^66^ ^]^ Figure S13 (Supporting Information) and Experimental Section contains more details on the resolution‐determining setup. Figure 4f shows the infrared images unveiled by the see‐through upconversion goggles. Real‐time, uniform, large‐area infrared patterns defined by the shadow mask can be precisely restored to sight through the semi‐transparent window, leveraging the high‐resolution image quality of OUD based on disordered molecules. Overall, a dual audio‐visual mode of infrared signal transmission has been realized in the compact upconversion goggles for wearable electronics. Beyond previous literature focused on the cardiac cycle required limited response speed, the electronic readout was explored by synchronizing the photocurrent with the light source at the acoustic frequency. We expect that this straightforward operation on the high‐resolution, high‐frequency infrared signal can inspire novel head‐mounted displays in the near future.

## 3 Conclusion

Preserving sufficient upconversion luminance based on the semi‐transparent device structure is challenging. Previous reports consuming single‐type charge carriers experienced substantial loss with limited upconversion efficiency. By redesigning the device structure via sandwiching the single‐component CGL between two EMLs full of localized charge carriers, all photoinduced charge carriers (i.e., electrons and holes) can be completely depleted. Thanks to the trivial spectrum overlap between the ClAlPc and the Ir(ppy)_2_(acac), nearly one‐third of the infrared photons are utilized without any optical lens or photomultiplication strategies; this value is, to the best of our knowledge, the best performance yet recorded. The ultrafast pump‐probe spectroscopy further explored the reason behind the doubled upconversion efficiency on the self‐dissociate ClAlPc. The transient photoexcitation dynamics suggested an efficient photocurrent generation from the ClAlPc neat layer. The lifetime of the charge carriers was prolonged by imposing the external electric field, facilitating the possibility of charge carrier recombination for light emission. In addition, unlike most studies focused on the optical imaging performance of the upconversion device, we opened up a new avenue of exploiting the electronic readout for high‐frequency signal transmission. We believe that the dual audio‐visual operation on the lightweight, see‐through, pixel‐free upconversion goggles offers an attractive alternative other than the existing imaging systems that are intrinsically bulky and opaque.

## 4 Experimental Section

### Device Fabrication

The crude materials including 1,4,5,8,9,11‐hexa‐azatriphenylene hexacarbonitrile (HATCN), 1,1‐bis‐(4‐bis(4‐methyl‐phenyl)‐amino‐phenyl)‐cyclohexane (TAPC), 9,9′‐diphenyl‐9H,9′H‐3,3′‐bicarbazole (BCzPh), Ir(ppy)_2_(acac), lithium fluoride (LiF), and the ITO‐coated substrate were acquired from Lumtec Corporation. 3′,3′″,3′″″‐(1,3,5‐triazine‐2,4,6‐triyl)tris(([1,1′‐biphenyl]−3‐carbonitrile)) (CN‐T2T) was purchased from Shine Materials. Before thin‐film deposition, all organic materials were sublimated twice in the temperature gradient purification system, and the substrates were soaked in an ultrasonic bath following the sequence of diluted detergent, deionized water, acetone, and isopropanol for 10 min each. Multilayered organic thin films were then successively developed in the thermal evaporator at a base vacuum level of 10^−6^ Torr. For the bipolar OUD, the device was configured with ITO (150 nm)/ HATCN (15 nm)/ TAPC (9 nm)/ BCzPh (10 nm)/ BCzPh: CN‐T2T: Ir(ppy)_2_(acac) (1:1:10% 30 nm)/ CN‐T2T (41 nm)/ CN‐T2T:10% Li_2_CO_3_ (12 nm)/ ClAlPc (30 nm)/ TAPC (37 nm)/ BCzPh (10 nm)/ BCzPh: CN‐T2T: Ir(ppy)_2_(acac) (1:1:10% 30 nm)/ CN‐T2T (41 nm)/ LiF (1 nm)/ Al (120 nm). However, for the semi‐transparent device, the top electrode was replaced with a multilayered thin film of CN‐T2T:10%Li_2_CO_3_ (41 nm)/ Ag:3% Cu (10 nm)/ WO_3_ (30 nm). Although the active area of each device was defined by the overlapping region of the ITO anode and metal cathode with 4 mm^2^ for the standard device structure, a shadow mask (2.082 mm^2^ opening) was used to define the active area during characterization in case the CGL outside the overlapping area contribute extra photocurrent.

### Characterization

The photon‐to‐photon upconversion efficiency can be calculated with $\;\eta_{p-p}\;\left(\%\right)\;=\frac{\int \frac{\lambda_{\mathrm{up}}I_{\mathrm{up}}\left(\lambda \right)}{\mathrm{hc}}d\lambda }{\frac{\lambda_{\mathrm{inc}}P_{\mathrm{inc}}}{\mathrm{hc}}}$ that describes the utilization rate of incoming infrared photons for visible photons.^[^ ^21^ ^]^ *h* and *c* are the Planck constant and the speed of light in the vacuum. *λ_up_* and *I_up_(λ)* are the upconversion visible wavelength and its corresponding intensity. *λ_inc_* and *P_inc_* are the incident infrared wavelength and the related irradiation power of interest. If enough band bending is satisfied, the leakage current under the dark contributes to the upconversion luminance under the infrared stimulus. However, in principle, the device can no longer distinguish a low‐intensity signal with upconversion luminance comparable to the leakage luminance, i.e., a poor signal‐to‐noise ratio. In this regard, we reported the maximum upconversion efficiency at which the device maintained linear response (LDR region) and excluded the leakage luminance to conform to the upconversion luminance in response to the infrared stimulus. The same practice was adopted on the OUD managing single‐type charge carriers in the Supporting Information for consistency. The photoluminescence spectrum, transmittance spectrum, absorption spectrum, EQE spectrum, specific detectivity spectrum, brightness, and current density were recorded according to the previous practice.^[^ ^26^ ^]^ The *AVT* can be calculated by the equation of $\mathrm{AVT}\left(\%\right)=\frac{\int T(\lambda )P(\lambda )S(\lambda )d\lambda }{\int P(\lambda )S(\lambda )d\lambda }$ where *T*, *P*, *S*, and *λ* represent the transmittance spectra, the human photopic response, solar photon flux, and the optical wavelength. A detailed description of the ultrafast pump‐probe spectroscopy can be found in Supporting Information. To evaluate the spatial resolution of the device, the system was set up on the operational stage of the optical microscope (Taiwan Microscope Enterprise). The infrared light source (M780L3, Thorlabs) was first collimated by the adapter (COP1‐B Olympus, Thorlabs) and propagated through the photomasks with a line‐shaped pattern (line spacing 3, 5, and 10 µm). The large‐area, semi‐transparent device revealed the infrared images, which were subsequently captured by the CCD camera for image resolution determination. Actual photos for the overall system setup can be found in the Supporting Information (Figure S13, Supporting Information).

## Conflict of Interest

The authors declare no conflict of interest.

## Supporting information

Supporting Information

Supplemental Video 1

## Data Availability

The data that support the findings of this study are available from the corresponding author upon reasonable request.
