# Supplementary material for: Semi‐Transparent, Pixel‐Free Upconversion Goggles with Dual Audio‐Visual Communication
Source: Adv Sci (Weinh). 2023 Sep 22;10(31):2302631. doi: 10.1002/advs.202302631 (PMC10625064; doi:10.1002/advs.202302631)
Supplement: Supplementary file 1 — Supporting Information [file ADVS-10-2302631-s001.pdf]

ADVANCED SCIENCE
Open Access

Supporting Information

for *Adv. Sci.*, DOI 10.1002/advs.202302631

Semi-Transparent, Pixel-Free Upconversion Goggles with Dual Audio-Visual Communication

Chun-Jen Shih, Chao-Yang Lin, Kai Chen, Nurul Ridho Al Amin, Dian Luo, I-Sheng Hsu, Abdul Khalik Akbar, Sajal Biring, Chih-Hsuan Lu, Bo-Han Chen, Shang-Da Yang, Jiun-Haw Lee* and Shun-Wei Liu**

Supporting Information

Semi-transparent, pixel-free upconversion goggles with dual audio-visual communication

Chun-Jen Shih, Chao-Yang Lin, Kai Chen, Nurul Ridho Al Amin, Dian Luo, I-Sheng Hsu, Abdul Khalik Akbar, Sajal Biring, Chih-Hsuan Lu, Bo-Han Chen, Shang-Da Yang, Jiun-Haw Lee,* Shun-Wei Liu**

C.-J. Shih, J.-H. Lee
Graduate Institute of Photonics and Optoelectronics and Department of Electrical Engineering, National Taiwan University, Taipei 10617, Taiwan.
E-mail: jiunhawlee@ntu.edu.tw

C.-J. Shih, N. R. A. Amin, D. Luo, I-S. Hsu, A. K. Akbar, S. Biring, S.-W. Liu
Organic Electronics Research Center and Department of Electronic Engineering, Ming Chi University of Technology, New Taipei City 24301, Taiwan.
E-mail: swliu@mail.mcut.edu.tw

C.-Y. Lin, K. Chen
Robinson Research Institute, Faculty of Engineering, Victoria University of Wellington, Wellington 6012, New Zealand

C.-H. Lu, B.-H. Chen, S.-D. Yang
Institute of Photonics Technologies, National Tsing Hua University, Hsinchu 30013, Taiwan
E-mail: sdyang@ee.nthu.edu.tw

K. Chen
MacDiarmid Institute for Advanced Materials and Nanotechnology, Wellington 6012, New Zealand.

K. Chen
The Dodd-Walls Centre for Photonic and Quantum Technologies, Dunedin 9016, New Zealand.

Steady-state photophysics

Apart from a long history of utilizing phthalocyanines to harness solar energy,[1] they were also studied by the large community of photodynamic therapy in oncology homologous with porphyrins and chlorins. Despite holding great promises, most phthalocyanines are hydrophobic and form stacked aggregates in an aqueous solution, making no exception for the ClAlPc molecules.[2]

To clearly understand our CGL, we discuss the steady-state photophysics of ClAlPc in this section, especially the intermolecular behavior between monomeric and aggregated conformations. Instead of disentangling the fundamental characteristics going over the molecular solution, we studied the compact solid-state thin film to conform with the CGL in the main text. The ClAlPc proportion was diluted in the CN-T2T inert matrix (triplet energy of 2.82 eV) to observe the dependence on the degree of aggregation (α_{agg}) and, consequently, the photophysical properties responsible for the neat layer. The representative absorption spectrum suggested that the ClAlPc monomer possessed two clear vibronic bands of interest (peaked at 636 nm and 706 nm in Figure S1). Both transition bands experienced a bathochromic shift as a function of average intermolecular distance (band maxima toward 670 nm and 766 nm, respectively), similar to that of metal-free phthalocyanine.[3] However, a broad degenerated Q-band composed of a new Gaussian stretching species at low frequency implied dimer formation in the 100% condensed film.[4] This highlights the pivotal role of molecular packing on the excitation transition,[5,6] especially on the highly symmetric ClAlPc molecule with planar conformation, which has been described in our previous morphological model.[7]

Under the excitation stimulus close to the Q-band edge (wavelength at 650 nm), photoluminescence spectra peaked at 711 nm can be discovered on low-concentration samples in agreement with the ClAlPc monomer dispersed in the solution (inset in Figure S1b).[2] This near-infrared emission decreased by the presence of the neighboring dimers with intimate π-contacts and can be attributed to the non-radiative relaxation pathways.[3] Strategies have been proposed to address this aggregate quenching issue on other chromophores with sterically hindered side chain decoration.[8] Here, we note that the possibility of dimer fluorescence has been excluded for the following reasons. First, the fluorescence emission followed a non-radiative self-quenching process with no new species observed in the spectra. Second, ClAlPc demonstrated a small Stokes shift with substantial overlap between the absorption and emission spectra to a large extent. The re-absorption effect can be reinforced when two monomers come to close contact. Besides, the decay profile can be readily described by considering the re-absorption effect solely on sulfonated ClAlPc in the literature.[4]

Upon examining the excitation wavelengths (monitor at 711 nm), concentration-dependent quenching progression was found again with two clear transition peaks (one at 630 nm and around 711 nm for the other) in line with the absorption spectra. The great similarity between the 1% solid-state thin film and the result retrieved from the solution sample supported our finding on the ClAlPc monomeric form by diluting the molecule in the CN-T2T matrix and was not sensitive to the detail of the molecular structure of the matrix host but the proximity of the ClAlPc monomer between each other. In brief, the intrinsic photophysical properties of the ClAlPc monomer were explored by diluting it into a wide bandgap inert matrix. Using this approach, we clarified that the strong intermolecular coupling between monomers could describe the low-frequency vibronic band of ClAlPc neat film. We harvested the incoming infrared photon from these representative transition states throughout the study with an excitation wavelength (780 nm to be exact) longer than the monomeric radiative states.

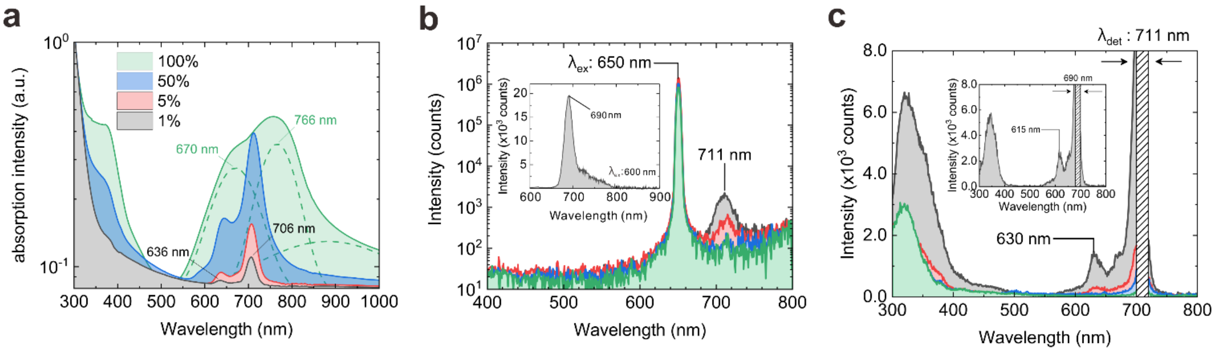

Figure S1. Photophysical analysis on diluted thin films. **a**) Absorption spectra, **b**) photoluminescence spectra, and **c**) excitation spectrum of ClAlPc diluted in CN-T2T solid state thin films. All figures share the same legend in **a**. The absorption spectrum of 100% ClAlPc neat film is fitted with three Gaussian distributions (green dash lines). The 650 nm spike in the photoluminescence spectra is the excitation source (inset shows the profile of the ClAlPc dispersed in dichloromethane, 10^{-5} M). The detection wavelength is set at 711 nm in the excitation spectra while 690 nm for the ClAlPc solution sample in the inset. We skip the profile around the radiative states to avoid damage from the high-intensity luminance in the excitation spectra (fill with shade lines).

Theoretical device modeling

In this section, we analyzed the passive optical characteristics of the OUD devices based on the transfer matrix method (TMM).[9,10] The depth mapping of optical field distribution in the wavelength range of 300 nm to 900 nm inside the OUD devices was demonstrated in Figure S2. A clear periodic profile can be discovered stretching the visible spectrum, i.e., 400 nm to 650 nm, suggesting the incident light is mainly affected by the waveguide effect instead of the absorption from the stacked thin films. Namely, visible light travels freely inside the OUD design with minimum re-absorption loss. However, a different profile was discovered in the ultraviolet and near-infrared wavelength due to the selective absorption bands of ClAlPc in

these regions (cf. Figure 1a). The periodic distribution was significantly suppressed when we focused on the ClAlPc Q-band (around 700-800 nm in wavelength). We further analyzed the field distributions of two represented monochromic wavelengths (Fig. 2d-f), which were the main peak of the upconversion luminance at 523 nm and the target signal at 780 nm. For all kinds of OUD structures, the optical field intensity of the 523 nm signal exhibited a depth-dependent periodic waveform. In comparison, the intensity of the 780 nm signal decayed after traveling through the ClAlPc layer. To explore the re-absorption effect of ClAlPc on the upconversion luminance, we replaced the ClAlPc charge generation layer with the wide bandgap TAPC in the device structure (solid spheres). A limited re-absorption effect was discovered on the main peak of the upconversion luminance at 523 nm. Therefore, we can conclude that ClAlPc performed as a wavelength-selective absorber, acting a minimum optical interference with the upconversion luminance in the device structure constructed.

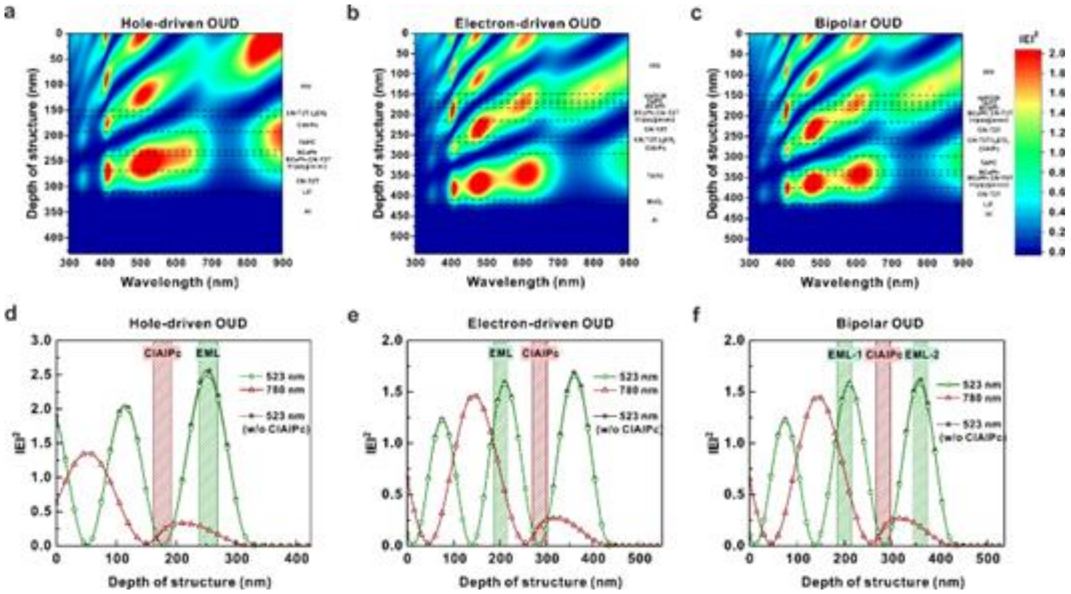

Figure S2. Depth distribution of the optical fields. **a)-c)** field mapping of the incident signal. **d)-f)** the field distribution of the two monochromatic wavelengths at 523 nm (olive) and 780 nm (wine). The solid black spheres are the field distribution replacing the ClAlPc with TAPC.

OUD managing single-type charge carriers

It is general to thermally deposit the OLED structure on top of the CGL concerning energy band alignment between the typical photoactive CGL and organic hole-transporting layer (HTL). It also avoided the damage of dissolving underlying layers when it came to solvent orthogonality for solution-processed CGLs. While most works chose CGL/ OLED structures based on these benefits, few studies focused on interfacial modification in the OLED/ CGL system.[11] In this section, we present both types of OUD for reference (All devices are driven with the same polarity as bipolar OUD). For readability, we classify the OUDs managing single-type charge carriers as *hole-driven OUD* and *electron-driven OUD* according to the charge

carrier supplied by the CGL.

For the *hole-driven OUD* (Figure S3), the device structure was configured as ITO (150 nm)/CN-T2T: 10%Li_2CO_3 (12 nm)/ ClAlPc (30 nm)/ TAPC (37 nm)/ BCzPh (10 nm)/ BCzPh: CN-T2T: $Ir(ppy)_2(acac)$ (1:1:10% 30 nm)/ CN-T2T (41 nm)/ LiF (1 nm)/ Al (120 nm). The device turned on at the bias voltage of 1.6V under infrared illumination with limited luminance recorded until the bias voltage of 10.4V (Figure S3a), spreading an operational range of 3.0-10.0V. However, the LDR was limited at 47 dB because the upconversion luminance was overwhelmed by the leakage current at the low-intensity region (Figure S3b). Higher LDR can be achieved by suppressing the dark current or enhancing upconversion luminance, as presented in the main text. The device achieved a specific detectivity approaching 10^{10} Jones with a similar responsivity spectrum but a slightly higher noise current than the bipolar OUD (Figure S3c). Although the device demonstrated similar EQE (Figure S3d) to the bipolar OUD presented in the main text, half of the carriers (electrons) recombined at the ITO anode, accounting for a substantial loss to the luminescent efficiency (Figure S3e). Overall, the hole-driven device achieved an upconversion efficiency of around 13.97-14.53% at 10.0V, depending on the incident infrared intensity.

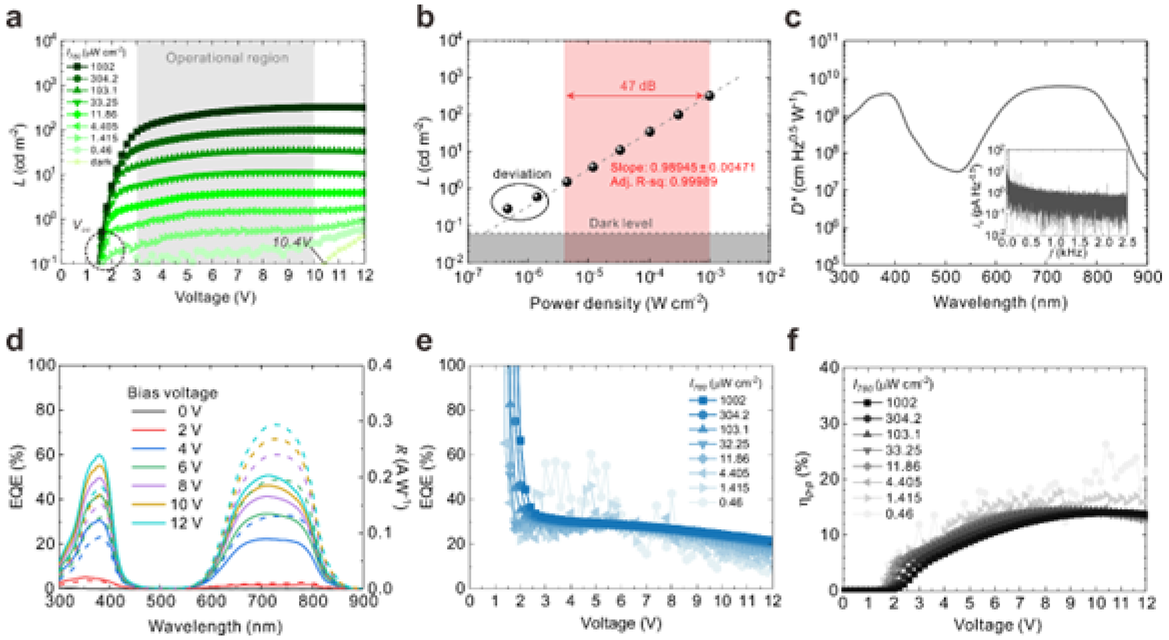

Figure S3. Hole-driven OUD under various infrared stimulus densities. **a**) Bias-dependent upconversion luminance (L) of the device under various infrared power densities. **b**), Upconversion luminance of the device driven at 10.0V under various infrared power densities (black spheres). The red area demonstrates the linear fitting result— the adjusted R square value describes the mean square deviation of the fitting. The grey dash line is the ideal case guide for the eye. The shaded grey level tells the leakage luminance under 10.0V without an infrared stimulus. **c**) Specific detectivity (D^*) spectra of the device driven at 10.0V. Inset shows the noise current (i_n) of the device recorded at the same bias. **d**) External quantum efficiency (EQE, solid lines) and responsivity (R, dash lines) spectra of the device under various bias voltages.

e) Bias-dependent electroluminescent EQE of the device under various infrared power densities. The large deviations approaching V_{on} are excluded for clarity. **f**) Bias-dependent photon-to-photon upconversion efficiency (η_{p-p}) of the device under various infrared power densities.

For the *electron-driven OUD* (Figure S4), the device structure was deployed as ITO (150 nm)/ HATCN (15 nm)/ TAPC (9 nm)/ BCzPh (10 nm)/ BCzPh: CN-T2T: Ir(ppy)$_2$(acac) (1:1:10% 30 nm)/ CN-T2T (41 nm)/ CN-T2T: 10%Li$_2$CO$_3$ (12 nm)/ ClAlPc (30 nm)/ TAPC (110 nm)/ MoO$_3$ (15 nm)/ Al (120 nm). We extended the distance between the EML and the reflective cathode by increasing the TAPC thickness to match the optimal light out-coupling rate of the upconversion luminance. Therefore, a leakage luminance was recorded until the bias voltage of 17.8V under dark, leading to an expanded operational region of 5.0-15.0V. Although the suppressed dark level increased the LDR to 57 dB (Figure S4b) and specific detectivity exceeding 10^{10} Jones (Figure S4c), once again, half carriers (holes) recombined at the aluminum cathode, causing a substantial loss in terms of current efficiency (Figure S4e). Overall, the electron-driven device achieved a slightly higher upconversion efficiency of around 13.60-15.35% at 15.0V (Figure S4f).

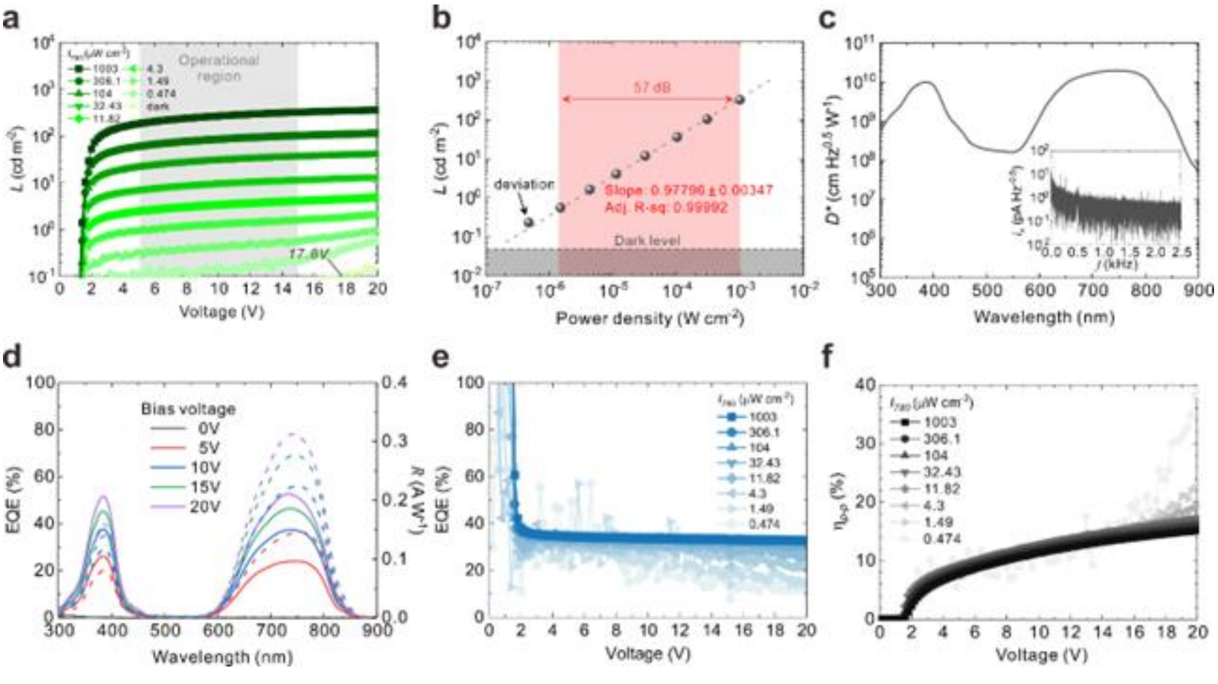

Figure S4. Electron-driven OUD under various infrared stimulus densities. **a**) Bias-dependent upconversion luminance (L) of the device under various infrared power densities. **b**) Upconversion luminance of the device driven at 15.0V under various infrared power densities (black spheres). The red area demonstrates the linear fitting result— the adjusted R square value describes the mean square deviation of the fitting. The grey dash line is the ideal case guide for the eye. The shaded grey level tells the leakage luminance under 15.0V without an infrared stimulus. **c**) Specific detectivity (D^*) spectra of the device driven at 15.0V. Inset shows the noise current (i_n) of the device recorded at the same bias. **d**) External quantum efficiency (EQE, solid lines) and responsivity (R, dash lines) spectra of the device under various bias voltages. **e**) Bias-dependent electroluminescent EQE of the device under various infrared power densities.

The large deviations approaching V_{on} are excluded for clarity. **f**) Bias-dependent photon-to-photon upconversion efficiency (η_{p-p}) of the device under various infrared power densities.

Single-Component Photodetector

In this section, we characterize the single-component CGL ClAlPc with OPD structures that encompass the external quantum efficiency (EQE) and responsivity (R) regarding the 780 nm monochromatic stimulus, dark current density (J_{dark}) as well as noise current (i_n) under external bias, linear dynamic range (LDR) attenuating the incoming light intensity, and a most important figure of merit above all, the specific detectivity (D^*). In general, D^* tells the sensitivity to differentiate the incident signal from the noise contribution and can be quantitatively figured by noise equivalent power ($NEP = i_n/R$) of the photodetector.[12]

The single-component OPD device is constructed with ITO (150 nm)/ CN-T2T (40 nm)/ CN-T2T:10%Li_2CO_3 (10 nm)/ ClAlPc (30 nm)/ TAPC (90 nm)/ MoO_3 (15 nm)/ Al (120 nm), following the stacking layers between two emission layers of the bipolar OUD in the main text. The sizeable energetic level difference at the anode and cathode interface guarantees limited dark current in the absence of infrared stimulation. As revealed in Figure S5, a suppressed dark current density of 1.4×10^{-8} A cm^{-2} as well as a suppressed noise current of several fA $HZ^{-1/2}$ can be achieved at +3.0V, suggesting a clear diode characteristic of the OPD device. The device can track the infrared power density down to submicron watt per centimeter square in linear, similar to that observed in bipolar OUD (Figure 2c in the main text). The OPD followed a precise wavelength selectivity toward the incident light in line with the absorption spectrum of the ClAlPc neat film (Figure 1a). Overall, the single-component OPD demonstrated a decent specific detectivity exceeding 10^{12} Jones (cm $Hz^{-1/2}$ W^{-1}) at the wavelength of 780 nm.

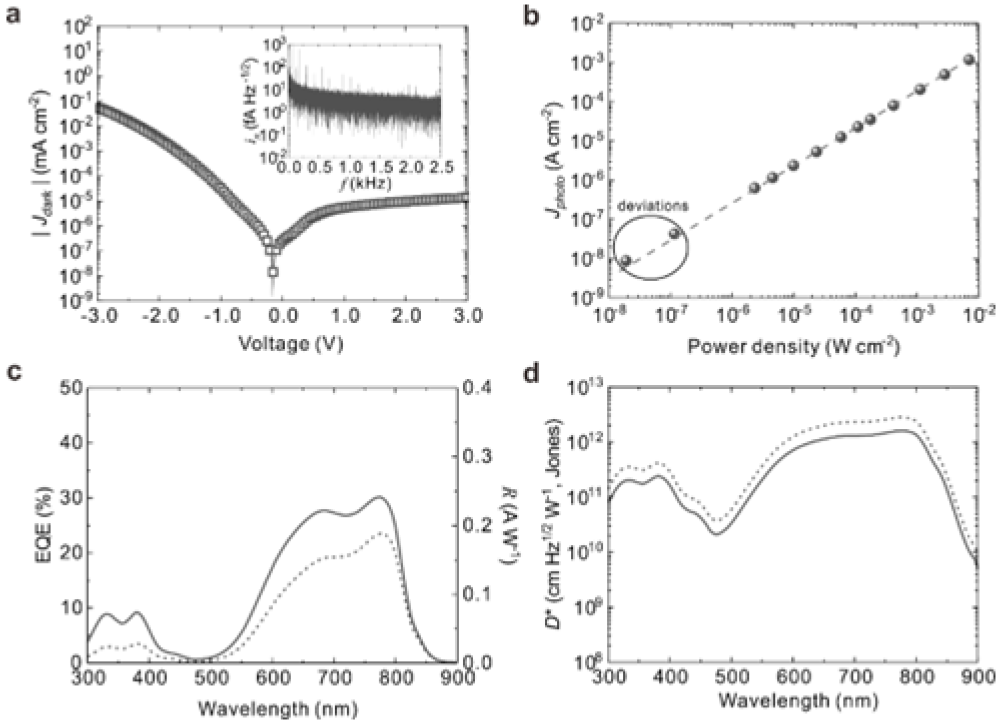

Figure S5. ClAlPc single-component OPD. **a**) Absolute dark current density ($|J_{dark}|$) of the device under different bias voltage. Inset shows the noise current (i_n) of the device recorded at +3.0V. **b**) The photocurrent density (J_{photo}) of the device (spheres) driven at +3.0V under various power densities of infrared stimulus. The grey dash line is the ideal case in linear. **c**) External quantum efficiency (EQE, solid line) and responsivity (R, dash line) spectra of the device driven at +3.0V. **d**) Specific detectivity (D^*) spectra of the device driven at +3.0V. The values are deduced from noise current (solid line by $D^* = R\sqrt{A\delta f}/i_n$) and dark current (dash line by $D^* = R/\sqrt{2qJ_{dark}}$), respectively.

Pump-Probe Spectroscopy

In this section, we will introduce the ultrafast pump-probe spectroscopy system for transient absorption (TA) measurement. The system started with a ytterbium-based laser (Light conversion Pharos) that produces fundamental pulses with a pulse duration of 240 fs, energy of 800 μJ, a center wavelength at 1030 nm, and a repetition rate of 12.5 kHz. We divided the fundamental pulses into two equivalent parts as the pump and probe for the spectroscopy and directed them through the double pass multiple plate compression (DPMPC)[13] along with a sequential multiple plate compression (MPC)[14-15] setup to realize ultrashort pulse compression. Thereafter, the compressed pulses passed through a 980 nm short-pass filter to prevent sample damage from the strong fundamental components. As a result, the probe spectrum spaned from 530 nm to 980 nm (Figure S6) at 20 dB level; the pump possessed a transform-limited pulse width of 14.28 fs when it was filtered with a 760 nm long-pass filter and a 900 nm short-pass filter to match the absorption spectrum of ClAlPc.

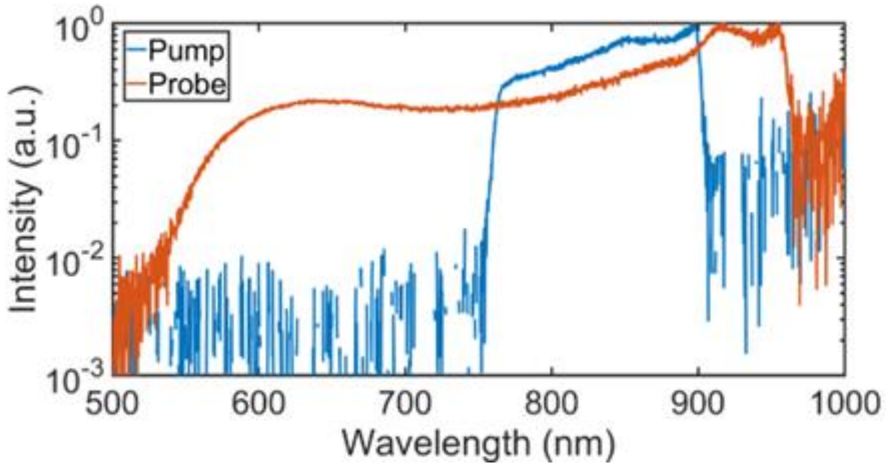

Figure S6. Spectra of pump and probe in ultrafast pump-probe spectroscopy. The probe spans from 530 nm to 980 nm beyond 20 dB level; pump is filtered in the range from 760 nm to 900 nm to match the absorption spectrum of ClAlPc.

In the optical path of the pump, an optical chopper synchronized with the laser at half of the laser repetition rate for the measurement of the sequential ground state and excited state transmission of the probe. We used a half-wave plate and a wire-grid polarizer to fine-tune the energy and polarization of the pump for adequate photoexcitation of the sample. By capturing the back-reflection of light from a photodiode, the laser shot was indexed. Finally, the pump beam was focused onto the sample as a spot of 50 μm diameter by a concave mirror.

We directed the probe through a motorized linear translation stage, which varied the delay between pump and probe, and overlapped the probe with the pump beam by focusing it to a spot of 20 μm onto the sample with a parabolic mirror. Uniform photoexcitation of the probe was guaranteed under such beam size ratio of pump and probe. Later, we recollimated and guided the probe, which was transmitted through or reflected by the sample depending on the transparency of the sample, through a home-built spectrometer equipped with a high-speed capturing CCD (Glaz Linescan II, Syntronic with S11639-01 CMOS, Hamamatsu) for high-speed data acquisition.

With this scheme, we can perform TA spectroscopy on both the thin film samples and the OPD device. Figure S7a displays the TA map of ClAlPc thin film sample, demonstrating the GSB signal around 785 nm, which corresponds to the absorption peak of ClAlPc[16] (cf. Figure 3c). Bilayer ClAlPc/TAPC displays similar responses as ClAlPc (Figure S7b), indicating no energy and charge transfer between ClAlPc and TAPC under the bilayer structure. The TA map of OPD under external bias of 0V (Figure S7c) and 5V (Figure S7d) show features of long-lived charge carriers, while the latter exhibits an obvious electroabsorption screening feature. The evolution of TA spectra along different delay times of each sample can be found in Figure S8.

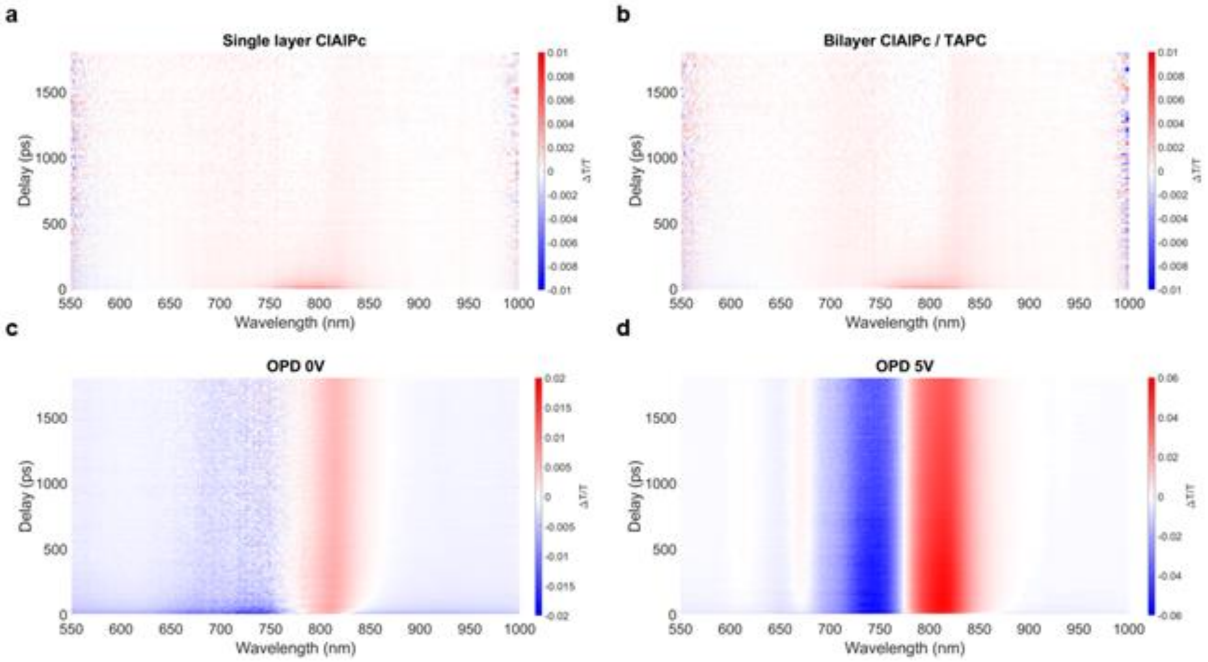

Figure S7. TA maps of **a**) single layer ClAlPc, **b**) bilayer ClAlPc/TAPC, **c**) OPD device under the bias voltage of 0V, and **d**) OPD device under the bias voltage of 5V.

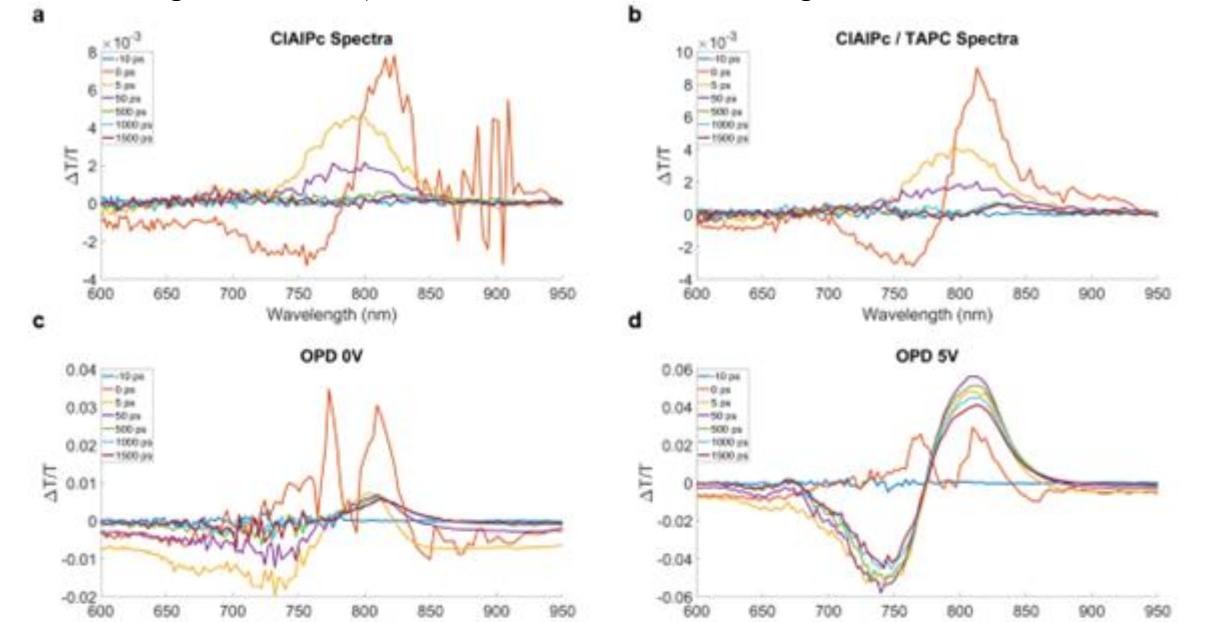

Figure S8. TA spectra at different delay time of **a**) single layer ClAlPc, **b**) bilayer ClAlPc/TAPC, **c**) OPD under 0V, and **d**) OPD under 5V. The oscillation structures observed in spectra at 0 ps are due to the nonlinear coherent artifact from the well overlap of pump and probe beam in the temporal and spatial domains.

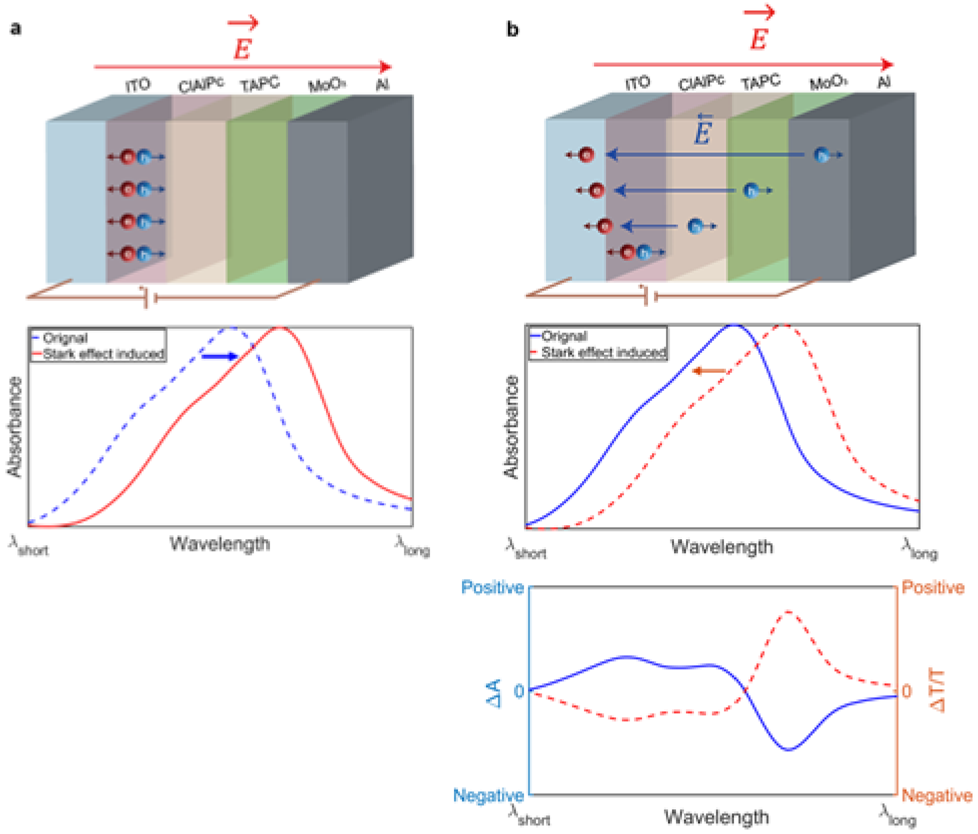

Figure S9. Screening of the electroabsorption spectrum due to free charge generation. **a**) Top: An external electric field (orange solid arrow) is applied on the ClAlPc OPD device. Bottom: The field shifts the absorption spectrum of ClAlPc (blue dash line) to a longer wavelength (orange solid line) before exciton dissociates due to the infrared illumination. **b**) Top: Infrared illumination dissociates excitons into free electrons and holes; these free carriers introduce an electric field (blue solid arrow) against the external field, reducing the Stark effect. Middle: The absorption spectrum blue shifts from the longer wavelength (orange dash line) towards the original spectrum (blue solid line) due to screening of electroabsorption. Bottom: Absorption change (blue solid line) and normalized reflection change (orange dash line) due to screening of electroabsorption.

Additionally, we conducted TA measurements on the bipolar OUD device under forward bias conditions. The obtained results reveal a similar electroabsorption screening effect as observed in the case of the OPD device. Specifically, a negative signal emerges at shorter wavelengths within a positive signal at longer wavelengths shortly after excitation. The kinetics of these positive and negative features are depicted in Figure S10, indicating similar photocurrent generation kinetics for both the OUD and OPD devices.

While the results obtained from the bipolar OUD are representative, it is important to note that our experiment primarily focused on investigating the exciton and photocurrent generation dynamics in the ClAlPc materials and active layer. These processes in organic optoelectronic materials and devices can be effectively probed using ultrafast spectroscopy, which operates in sub-picosecond (ps) to nanosecond (ns) time scales[17-23]. However, it is worth mentioning that

charge dynamics, including charge transport[24], bimolecular recombination[24,25], up-conversion, and subsequent light emission processes[26,27], occur beyond the nanosecond timescale, which falls outside the scope of ultrafast spectroscopy.

Given that OPD is a photoactive region of OUD device, it is reasonable to assume that the ultrafast processes observed in the OPD device will also occur in the OUD device and indeed, we have observed similar spectral features and kinetics between both devices. Consequently, our findings suggest that investigating the OPD device using TA measurements provides a valid approach to studying exciton and photocurrent dynamics for OPD applications.

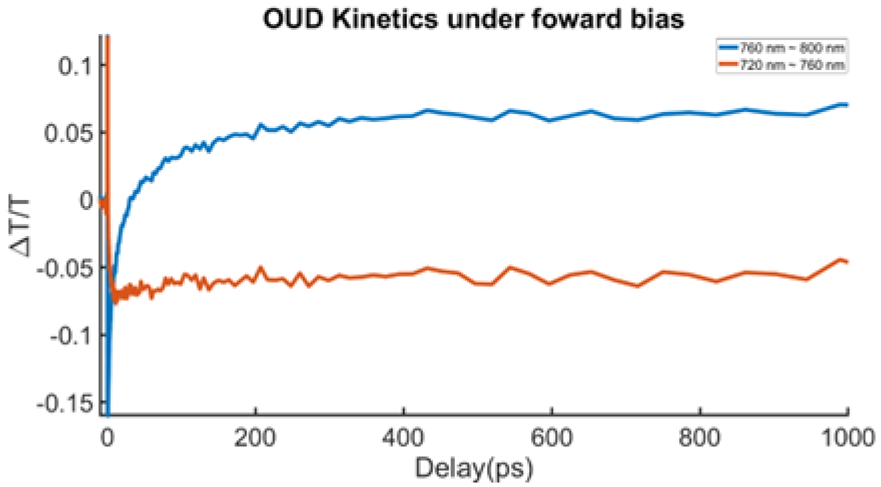

Figure S10. The kinetics of OUD under forward bias were studied within two wavelength ranges: 720 nm to 760 nm and 760 nm to 800 nm.

Dual audio-visual transmission

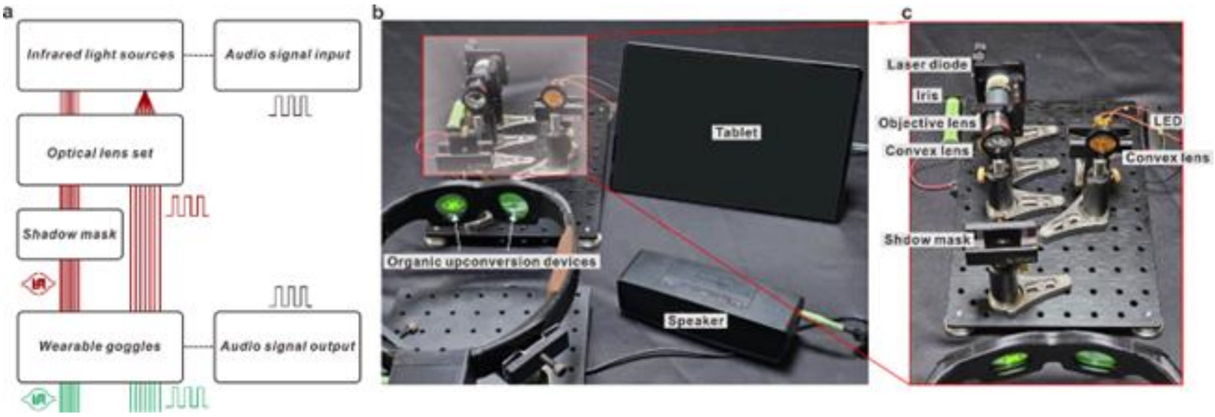

Figure S11. a) The schematic illustration of the overall system setup. **b**) As the left device reveals the infrared images defined by the shadow mask, the right one broadcasts the audio signal by synchronizing with the light source frequency. **c**) The enlarged image of the infrared signal processing.

Resolution determination

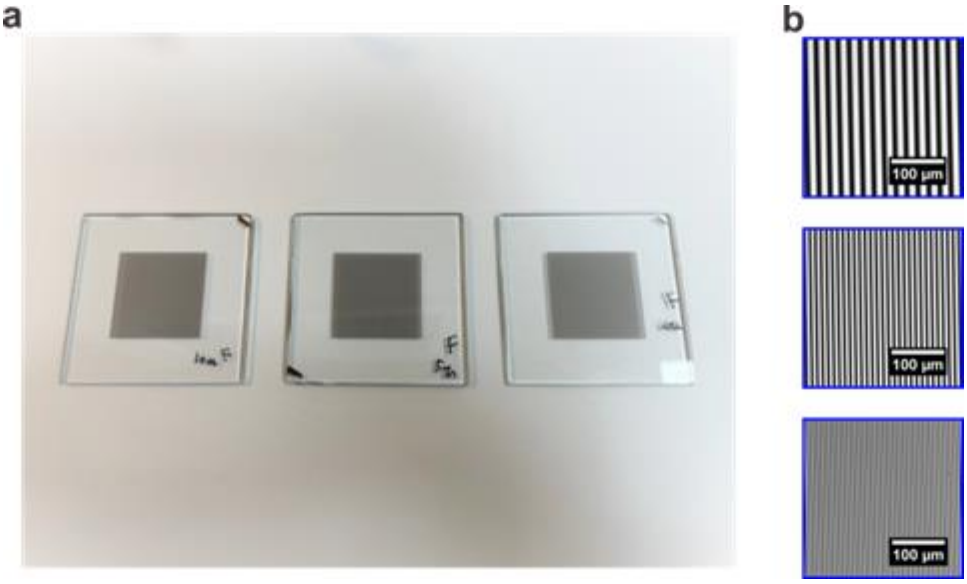

Figure S12. Supplementary information on the line-shaped photomask. The width of the narrow channel was ascertained by the optical microscope (BX53F2, Olympus) and calibrated by the image processing program (ImageJ). **a**) The photomask image. **b**) The photomask images captured by the optical microscope.

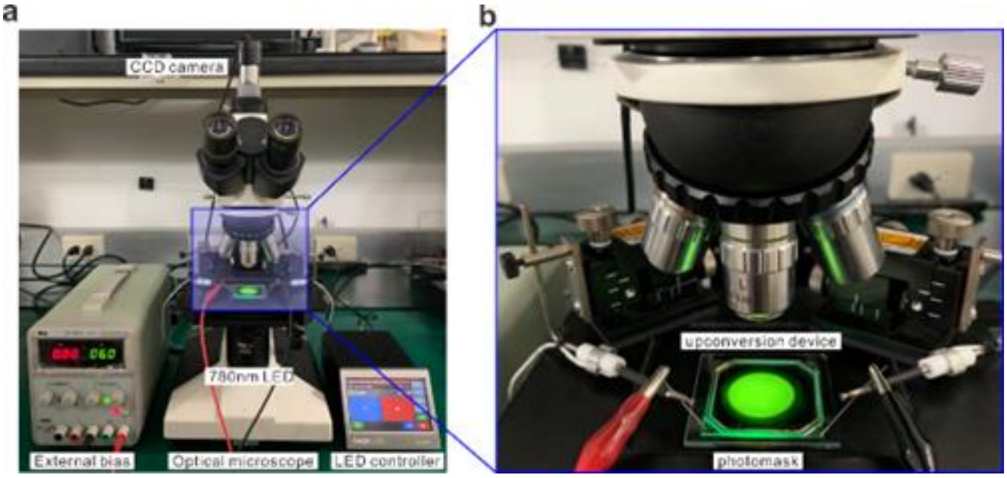

Figure S13. Supplementary information on the resolution determination process. **a**) The actual images of the resolution setup. **b**) The enlarged image of the operational stage. The infrared line stripes were defined by the infrared light (power density of around 1.0 mW cm^{-2}) propagating through the narrow spacing of the photomask (Figure S11). The infrared image was upconverted by the semi-transparent device (driven at 6.0V) placed on top of the photomask.

References

[1] C.-F. Lin, M. Zhang, S.-W. Liu, T.-L. Chiu, J.-H. Lee, *Int. J. Mol. Sci.* **2011**, *12*, 476.

[2] Italo Rodrigo Calori, Antonio Claudio Tedesco, *Dyes Pigm.* **2020**, *173*, 107940.

[3] B. W. Caplins, T. K. Mullenbach, R. J. Holmes, D. A. Blank, *J. Phys. Chem. C* **2015**, *119*,

27340.
[4] S. Dhami, A. J. deMello, G. Rumbles, S. Bishop, D. Phillips, A. Beeby, *Photochem. Photobiol.* **1995**, *61*, 341.
[5] D. Placencia, W.-N. Wang, R. Clayton Shallcross, K. W. Nebesny, M. T. Brumbach, N. R. Armstrong, *Adv. Funct. Mater* **2009**, *19*, 1913.
[6] M.-S. Choi, S. Chae, H. J. Kim, J.-J. Kim, *ACS Appl. Mater. Interfaces* **2018**, *10*, 25614.
[7] M. Wang, Y.-Z. Li, H.-C. Chen, C.-W. Liu, Y. Chen, Y.-C. Lo, C.-S. Tsao, Y.-C. Huang, S.-W. Liu, K.-T. Wong, B. Hu, *Mater. Horiz.* **2020**, *7*, 1171.
[8] R. O. Al-Kaysi, T.-S. Ahn, A. Müller, C. J. Bardeen, *Phys. Chem. Chem. Phys.* **2006**, *8*, 3453.
[9] Leif, L. S. Roman, Olle Inganäs, *J. Appl. Phys.* **1999**, *86*, 487.
[10] P. Peumans, Aharon Yakimov, S. R. Forrest, *J. Appl. Phys.* **2003**, *93*, 3693.
[11] C.-H. Yuan, C.-C. Lee, C.-F. Liu, Y.-H. Lin, W.-C. Su, S. Lin, K.-T. Chen, Yan De Li, Wen Shin Chang, Y.-Z. Li, T.-H. Su, Y.-H. Liu, S.-W. Liu, *Sci. Rep.* **2016**, *6*, 32324.
[12] Y. Fang, A. Armin, P. Meredith, J. Huang, *Nat. Photonics* **2018**, *13*, 1.
[13] B. Chen, J. Su, J.-Y. Guo, K. Chen, S.-W. Chu, H.-H. Lu, C.-H. Lu, S.-D. Yang, *Front. Photonics* **2022**, *3*, 937622.
[14] C.-H. Lu, W.-H. Wu, S.-H. Kuo, J.-Y. Guo, M.-C. Chen, S.-D. Yang, A. W. Kung, *Opt. Express* **2019**, *27*, 15638.
[15] Y.-C. Cheng, C.-H. Lu, Y.-Y. Lin, A. W. Kung, *Opt. Express* **2016**, *24*, 7224.
[16] V. Gulbinas, M. Chachisvilis, A. Persson, S. Svanberg, V Sundstroem, *J. Phys. Chem.* **1994**, *98*, 8118.
[17] Q. Cui, J. He, M. Z. Bellus, Mirzojamshed Mirzokarimov, T. Hofmann, H.-Y. Chiu, M. Antonik, D. He, Y. Wang, H. Zhao, *Small* **2015**, *11*, 5565.
[18] Q. Cui, F. Ceballos, N. Kumar, H. Zhao, *ACS Nano* **2014**, *8*, 2970.
[19] T. Virgili, G. Cerullo, L. Lüer, Guglielmo Lanzani, Christoph Gadermaier, D. A. Bradley, *Phys. Rev. Lett.* **2003**, *90*, 247402
[20] J. Cabanillas-Gonzalez, T. Virgili, A. Gambetta, Guglielmo Lanzani, T. D. Anthopoulos, M. de, *Phys. Rev. Lett.* **2006**, *96*, 106601.
[21] Rokas Jasiūnas, H. Zhang, J. Yuan, X. Zhou, D. Qian, Y. Zou, Andrius Devižis, Juozas Sulskus, F. Gao, Vidmantas Gulbinas, *J. Phys. Chem. C* **2020**, *124*, 21283.
[22] C. Zenz, W. Graupner, G. Cerullo, Guglielmo Lanzani, M. Nisoli, E. List, F. Meghdadi, G. Leising, S. De Silvestri, *Opt. Mater.* **1999**, *12*, 273.
[23] W. Graupner, G. Cerullo, Guglielmo Lanzani, M. Nisoli, E. List, G. Leising, S. De

Silvestri, *Phys. Rev. Lett.* **1998**, *81*, 3259.

[24] I. A. Howard, R. Mauer, M. Meister, LaquaiF., *J. Am. Chem. Soc.* **2010**, *132*, 14866.

[25] A. J. Barker, K. Chen, J. M. Hodgkiss, *J. Am. Chem. Soc.* **2014**, *136*, 12018.

[26] J. Zhao, S. Ji, H. Guo, *RSC Adv.* **2011**, *1*, 937.

[27] Y. Sasaki, Mio Oshikawa, Pankaj Bharmoria, Hironori Kouno, A. Hayashi-Takagi, M. Sato, Itsuki Ajioka, N. Yanai, Nobuo Kimizuka, *Angew. Chem. Int. Ed.* **2019**, *58*, 17827.
